# Thermal decomposition characteristics of foundry sand for cast iron in nitrogen atmosphere

**DOI:** 10.1098/rsos.181091

**Published:** 2018-12-12

**Authors:** Qingwei Xu, Kaili Xu, Xiwen Yao, Jishuo Li, Li Li

**Affiliations:** Key Laboratory of Ministry of Education on Safe Mining of Deep Metal Mines, School of Resources and Civil Engineering, Northeastern University, Shenyang 110819, People's Republic of China

**Keywords:** thermal decomposition, foundry sand, cast iron, volatile release parameter, activation energy

## Abstract

Sand casting, currently the most popular approach to the casting production, has wide adaptability and low cost. The thermal decomposition characteristics of foundry sand for cast iron were determined for the first time in this study. Thermogravimetry was monitored by simultaneous thermal analyser to find that there was no obvious oxidation or combustion reaction in the foundry sand; the thermal decomposition degree increased as the heating rate increased. There was an obvious endothermic peak at about 846 K due to the transition of quartz from *β* to *α* phase. A novel technique was established to calculate the starting temperature of volatile emission in determining the volatile release parameter of foundry sand for cast iron. Foundry sand does not readily evaporate because its volatile content is only about 2.68 wt% and its main components have high-temperature stability. The thermal decomposition kinetics parameters of foundry sand, namely activation energy and pre-exponential factor, were obtained under kinetics theory. The activation energy of foundry sand for cast iron was small, mainly due to the wide temperature range of thermal decomposition in the foundry sand.

## 1. Introduction

Foundry is a metal hot working technology that was long ago mastered by human societies, and currently the main method of casting moulding in the machine building industry [1,2]. The industries and national economy in China have rapidly developed [3–6]. China's founding industry is growing quickly, but improving the quality of its castings is yet necessary. A large foundry industry promotes economic development, but also may substantially pollute the environment [7–9] and endanger the lives of its personnel [10,11], and countermeasures should be adopted to eliminate the serious results [12,13]. Castings produced by sand casting account for approximately 90 wt% of the total casting output [14]. The local temperature of foundry sand exceeds 1773 K during alloy casting [15], which makes it extremely challenging to measure temperature changes in the sand during casting. Information regarding the thermal characteristic of foundry sand can be used to safeguard the production process while improving the casting quality.

Severe heat conduction phenomena occur when molten alloy is poured into the cavity of foundry sand [16]. Computer-aided numerical simulation is often used by sand casting researchers to better understand such phenomena [17]. Procast software can effectively simulate the stress, temperature and flow fields during the sand casting process [18,19]. Procast simulation information can be used to truncate the casting production cycle and determine certain parameters that are not easily obtained in the field. Zhang *et al*., for example, investigated the accuracy of a heat conduction model with the help of Procast software by comparison against analytic solutions [19]. Procast software can simulate the temperature field during the casting process, but it is not applicable to the thermal decomposition characteristic of foundry sand.

Thermal decomposition behaviour studies typically centre around coal, wood, rice straw, peanut shell, corncob and sewage sludge [20–25]. Wang *et al*. investigated the copyrolysis behaviour of Pingshuo coal using a thermogravimetric analyser to find that the addition of biomass facilitates pyrolysis and combustion of the coal [20]. Coronado *et al*. investigated the thermal characteristics of foundry sand dust to find that higher temperature and lower firing rate yield higher acid gas emissions [26]. There has been no study to date on the thermal decomposition behaviours of foundry sand for cast iron; the present study was conducted in an effort to fill this gap.

The starting temperature of volatile release must be known before determining the volatile release parameter corresponding to a mass loss rate up to 0.1 mg min^−1^ [20]. The sample mass in a thermal decomposition experiment is usually 5 mg [15], so the starting temperature of volatile emission corresponding to the mass loss rate is 2 wt% min^−1^. The main chemical component of foundry sand is SiO_2_, at up to 96.62 wt% [27]. The volatile content of foundry sand is very low and the volatile release parameter cannot be strictly calculated based on the concept. In this paper, a new method was proposed to calculate the starting temperature of volatile emission in determining the volatile release parameter of foundry sand, as confirmed by a case study.

Activation energy refers to the energy needed for molecules to move from the normal state to an active state that is prone to chemical reaction [24,25]. Activation energy effectually reflects the stability of the material. In the present study, the activation energy of foundry sand for cast iron was calculated under kinetics theory for the first time.

The main purpose of this study was to investigate the thermal decomposition characteristics of foundry sand for cast iron. The mass losses of the foundry sand at different heating rates were investigated and the volatile release parameter and activation energy of foundry sand were obtained. As mentioned above, a new method was established to calculate the starting temperature of volatile emission in determining the volatile release parameter of foundry sand and confirmed by a case study.

## 2. Material and methods

### 2.1. Foundry sand for cast iron

The foundry sand for cast iron that was used in this study was obtained from a foundry research institution in Shenyang, China. Prior to the thermal decomposition experiment, proximate analysis was carried out in a high-temperature environment (TRGF-8000 Automatic Proximate Analyser, Electronic and Technology Co. Ltd, Tianrun Hebi, China). The foundry sand is mainly constituted of ash (96.81 wt%), volatiles (2.68 wt%) and moisture (0.51 wt%).

### 2.2. Thermal decomposition experiment

The thermogravimetric experiment was conducted on a STA449 F3 sensitive thermal analyser (NETZSCH Group, Germany) in nitrogen atmosphere at heating rates of 30 and 40 K min^−1^. The temperature range of the thermal decomposition experiments was from 308 to 1473 K.

### 2.3. Volatile release parameter

The volatile release parameter was adopted to describe the thermal decomposition behaviours of foundry sand for cast iron. A larger volatile release parameter indicates better releasing characteristics and a more facile thermal decomposition reaction in the foundry sand sample. Assuming there is no water release in the initial phase and inorganic salt decomposition in the later stage, the volatile release parameter can be defined as follows [15]:

2.1 $$D=\frac{{\left(\text{d}w/d\tau \right)}_{\max}{\left(\text{d}w/d\tau \right)}_{\mathrm{mean}}}{(T_{s}-273)(T_{\max}-273)\Delta_{1/2}},$$

where *T_s_* denotes the starting temperature of volatile emission corresponding to the mass loss rate at 0.1 mg min^−1^ in K [20]; *T*_max_ denotes the temperature corresponding to the maximum mass loss rate in K; (*dw*/*dτ*)_mean_ denotes the mean mass loss rate of volatiles in wt% min^−1^; (d*w*/d*τ*)_max_ denotes the maximum mass loss rate of volatiles in wt% min^−1^ and Δ_1/2_ denotes the temperature range within the scope of (d*w*/d*τ*)/(d*w*/d*τ*)_max_ = 1/2 in K.

### 2.4. Kinetics theory

The Coats–Redfern method is typically used to describe the thermal decomposition of materials [20,28]. Based on the Arrhenius equation and the law of mass conservation of materials, the kinetics theory of the reaction is as follows:

2.2 $$-\ln\ln\;\left[\frac{-\ln’(1-\alpha )}{T^{2}}\right]=\frac{E}{RT}-\ln\ln\;\left[\frac{AR}{\beta E}\left(1-\frac{2RT}{E}\right)\right],$$

where *A* indicates the pre-exponential factor, min^−1^; *E* is the activation energy, kJ mol^−1^; *R* is the perfect gas constant, 8.314 kJ mol K^−1^; *β* is the heating rate, K min^−1^; and *T* is the thermal decomposition temperature, *K*.

The parameter *α* indicates the mass conversion ration of the thermal decomposition process [28],

2.3 $$\alpha =\frac{m_{0}-m_{t}}{m_{0}-m_{\infty }},$$

where *m*_0_, *m*_∞_ and *m_t_* indicate the starting mass of the sample, the ultimate mass of the sample and the mass of the sample at time *t*, respectively.

The monomial 2*RT*/*E* is much less than 1 and therefore can be omitted to simplify equation (2.2) as follows [25]:

2.4 $$-\ln\;\left[\frac{-\ln\;(1-\alpha )}{T^{2}}\right]=\frac{E}{RT}-\ln\;\left(\frac{AR}{\beta E}\right).$$

For the specific heating rate and temperature range, if the expression −ln[ −ln(1 − *α*)/*T*^2^] is plotted versus 1/*T* with a slope of *E*/*R*, then a straight line can be obtained with the intercept term −ln(*AR*/*βE*). The thermal decomposition kinetic parameters, that is, activation energy *E* and pre-exponential factor *A*, can be determined by equation (2.4).

## 3. Results and discussion

### 3.1. Thermal decomposition characteristics of foundry sand for cast iron

**Figure 1.**
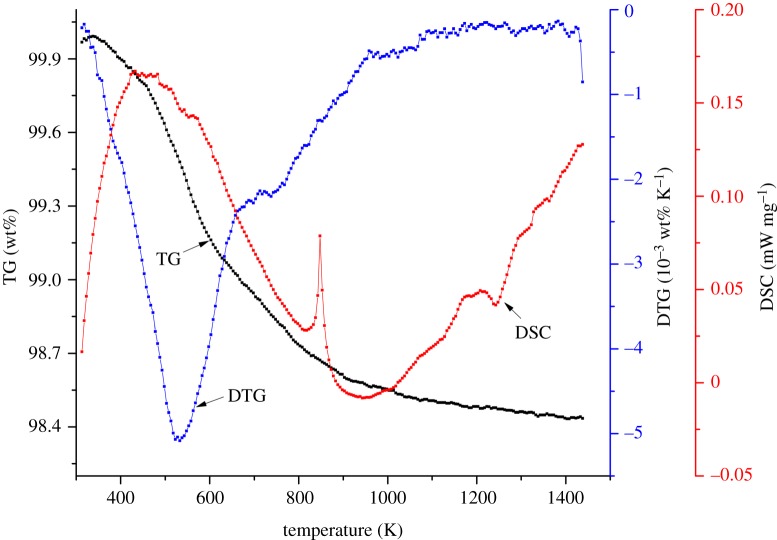
Thermal decomposition curves of foundry sand sample.

The mass of foundry sand for cast iron decreases gradually during the thermal decomposition process accompanied by decalescence or heat release. The thermal decomposition of the foundry sand sample in nitrogen at a heating rate of 30 K min^−1^ is shown in figure 1.

In figure 1, TG is short for ‘thermogravimetric', DTG is short for ‘derivative thermal gravimetric' and DSC indicates ‘differential scanning calorimetry'. The upward and downward DSC curves denote decalescence and exothermal activity, respectively. The thermal decomposition of foundry sand for cast iron can be roughly divided into three stages. The first stage of mass loss mainly occurred below 423 K, where the foundry sand mass slowly decreased. Although the DSC curve indicates an endothermic state in this stage, there is no endothermic peak. There is, however, an endothermic peak at 400 K in the DSC curve due to absorbed moisture volatilization [23]. The moisture content of foundry sand for cast iron is as low as 0.51 wt% in the sample that was used in this study, so there was no endothermic peak during the temperature rise stage.

The second stage of mass loss mainly took place from 423 to 673 K, where volatile devolatilization occurred. The TG curve sharply decreased during this stage and the corresponding DTG curve showed endothermic peaks mainly attributable to the thermal decomposition of the adhesives. The maximum mass loss rate of foundry sand for cast iron was −0.153 wt% min^−1^, and the corresponding peak temperature was 533 K. The mass loss of foundry sand for cast iron in this thermal decomposition stage contributed about 39 wt% to the total mass loss. In a previous study, foundry sand dust entered an endothermic state after about 473 K in an air atmosphere [26]. This was mainly due to the combustion or oxidation reaction of impurities in the foundry sand dust. The thermal decomposition process of foundry sand in this study can be attributed to the adhesives; there was no obvious combustion or oxidation reaction in the nitrogen atmosphere.

The third thermal decomposition stage of foundry sand for cast iron mainly occurred above 673 K. The remaining adhesives continued to decompose slowly and the mass loss rate of foundry sand was significantly reduced. There was an obvious endothermic peak at about 846 K due to the transition of quartz from *β* to *α* phase.

### 3.2. Thermal decomposition of foundry sand at different heating rates

**Figure 2.**
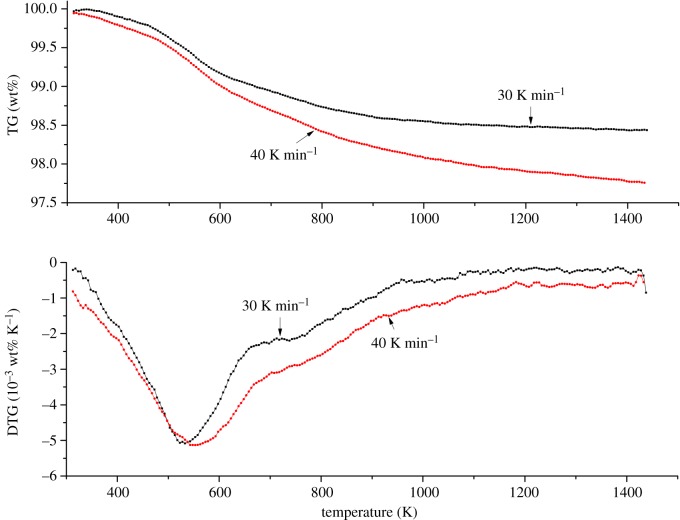
Thermal composition of foundry sand at heating rates of 30 and 40 K min^−1^.

The adhesives of foundry sand for cast iron have different thermal decomposition characteristics at different heating rates. The thermal composition characteristics of foundry sand for cast iron at heating rates of 30 and 40 K min^−1^ in nitrogen were shown in figure 2.

As shown in figure 2, the mass loss of foundry sand for cast iron increased as the heating rate increased. The mass loss of foundry sand was 1.57 wt% at a heating rate of 30 K min^−1^ and 2.24 wt% at a heating rate of 40 K min^−1^. Increasing the heating rate appears to enhance the thermal decomposition of foundry sand in a high-temperature environment. The mass loss of corncob also increases as the heating rate increases [25].

The main component of foundry sand is SiO_2_; the SiO_2_ content can be up to 96.62 wt% [27]. The mass loss of foundry sand is comparatively small in the high-temperature environment due to the high-temperature stability of SiO_2_, which also facilitates the sand casting moulding process. Here, the mass loss rate of foundry sand increased as the heating rate increased according to the DTG curve shown in figure 2. The maximum mass loss rate was −5.08 × 10^−3^ wt% K^−1^ at a heating rate of 30 K min^−1^ and −5.15 × 10^−3^ wt% K^−1^ at a heating rate of 40 K min^−1^. Again, increasing the heating rate appears to enhance the thermal decomposition of foundry sand in a high-temperature environment. The maximum mass loss rate of peanut shell also increases as the heating rate increases [24].

The peak temperature of the DTG curve of foundry sand also increased as the heating rate increased: the maximum peak temperatures were 533 and 556 K at heating rates of 30 and 40 K min^−1^, respectively. The thermal hysteresis phenomenon was mainly caused by the temperature gradient between the landmark and the sample, as well as between the inside and outside of the sample [25]. For a specialized temperature, a longer time will be needed for the smaller heating rate than the larger heating rate. The specific heat capacity of foundry sand is larger than air. When the environment temperature reaches the specialized temperature, the foundry sand sample of smaller heating rate will have longer time to transfer heat than the larger heating rate and more easily reach the specialized temperature. The temperature gradient of foundry sand sample of larger heating rate is bigger than the smaller heating rate. For the same heated condition, the higher environment temperature will be needed for the foundry sand sample of larger heating rate than the smaller heating rate. Therefore, the peak temperature of the DTG curve of foundry sand increased as the heating rate increased.

### 3.3. Volatile release parameter of foundry sand

The volatile release parameter of foundry sand can be obtained based on the thermal decomposition process. To determine the starting temperature of volatile emission *T_s_*, the mass loss rate of the sample must be at least 0.1 mg min^−1^ [20]. The mass of sample is usually 5 mg when carrying out a thermogravimetric experiment [15], so the starting temperature of volatile emission *T_s_* corresponds to the mass loss rate at 2 wt% min^−1^. The maximum mass loss rate of foundry sand for cast iron was 2.06 × 10^−1^ wt% min^−1^ in this study, so the starting temperature of volatile emission in determining the volatile release parameter of foundry sand cannot be achieved based on the previous method [15,20]. In this study, a novel technique was established to calculate the starting temperature of volatile emission in determining the volatile release parameter of foundry sand, shown as follows.

#### 3.3.1. Thermal composition process of rice husk

**Figure 3.**
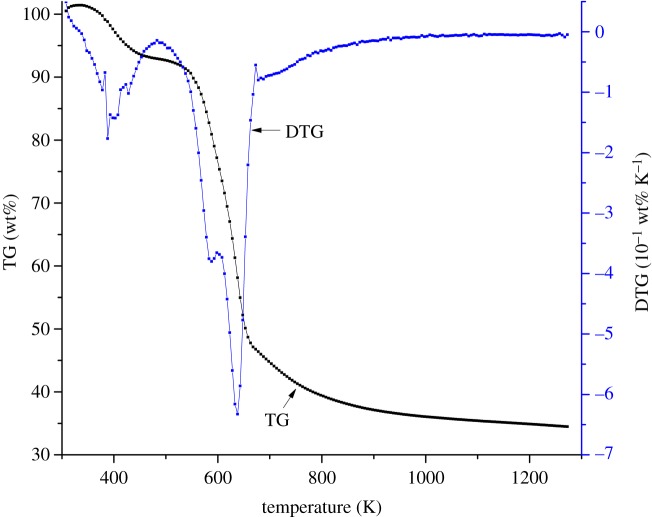
Thermal decomposition curves of rice husk.

The thermal composition process of rice husk was carried out at a heating rate of 40 K min^−1^ in a nitrogen atmosphere over a temperature from 308 to 1273 K, shown in figure 3.

The thermal decomposition of rice husk can also be roughly divided into three stages. The first stage of mass loss mainly occurred below 550 K, where the rice husk mass slowly decreased. The DTG curve has a downward peak at 381 K due to the absorbed moisture volatilization [23]. The evaporation of moisture in the rice husk makes a great contribution to the mass loss at temperatures below 550 K.

The second stage of mass loss mainly took place from 550 to 670 K, in which the maximum mass loss rate appears. The TG curve of rice husk sharply decreased during this stage and the corresponding DTG curve showed downward peak mainly attributable to the thermal decomposition of hemicellulose and cellulose [23]. The maximum mass loss rate of rice husk was −25.176 wt% min^−1^, and the corresponding temperature was 637 K. The mass loss of rice husk in this thermal decomposition stage contributed 66 wt% to the total mass loss.

The third thermal decomposition stage of rice husk mainly occurred above 670 K, and there was no remarkable mass loss in this stage. The remaining hemicellulose and cellulose continued to decompose slowly and the mass loss rate was significantly reduced.

#### 3.3.2. Volatile release parameter of rice husk

When the starting temperature of volatile emission *T_s_* corresponds to the mass loss rate at 2 and 3 wt% min^−1^, the volatile release parameters *D* of rice husk were first calculated according to equation (2.1), shown in table 1. Let Δ_1/2_ denote the temperature range from the starting temperature of volatile emission to the ending in this section.

**Table 1.** Volatile release characteristics of rice husk.

*T*_stv_ (wt% min^−1^)	*T_s_* (K)	*T*_max_ (K)	(d*w/*d*τ*)_max_ (wt% min^−1^)	(d*w/*d*τ*)_mean_ (wt% min^−1^)	△_1/2_ (K)	*D* (wt%^2^ min^−2^ K^−3^)	*f* (wt%^2^ min^−1^ K^−3^)
2	529	637	25.2	2.7	225	3.24 × 10^−6^	1.06 × 10^−4^
3	541				141	4.94 × 10^−6^	1.08 × 10^−4^
4	548				119	5.71 × 10^−6^	0.94 × 10^−4^

In table 1, the parameter *T*_stv_ denotes the threshold value that determining the starting temperature of volatile emission *T_s_*. The parameter *f* is a self-defining parameter which is related to the type of thermal decomposition material:

3.1 $$f=\frac{D}{T_{\mathrm{stv}}/m_{\mathrm{loss}}},$$

where *m*_loss_ denotes the sample's mass loss.

The mass loss of rice husk was 65.53 wt% in this study. As shown in table 1, the self-defining parameter *f* was almost invariant at about 10^−4^. That is to say, the self-defining parameter *f* for rice husk is 10^−4^.

To validate the proposed method for calculating the starting temperature of volatile emission in determining the volatile release parameter, the parameter *T*_stv_ of 4 wt% min^−1^ was also tested, shown in table 1. The results showed that the self-defining parameter *f* was also about 10^−4^, indicating that the proposed method for calculating the starting temperature of volatile emission in determining the volatile release parameter has validity.

The mass loss range of biomass was from 69 to 77 wt% at a heating rate of 20 K min^−1^ [25,29]. Sample mass loss of 70 wt% was selected in this study. The mass of thermal decomposition sample was 5 mg [15] and the parameter *T*_stv_ was 2 wt% min, so the monomial *T*_stv_/*m*_loss_ was 2.86 wt% min^−1^.

#### 3.3.3. Volatile release parameter of foundry sand in nitrogen atmosphere

The volatile release characteristics of foundry sand can be achieved based on the above analysis, shown in table 2.

**Table 2.** Volatile release characteristics of foundry sand.

*β* (K min^−1^)	*T*_stv_ (wt% min^−1^)	*m*_loss_ (wt%)	*T_s_* (K)	*T*_max_ (K)	(d*w/*d*τ*)_max_ (wt% min^−1^)	(d*w/*d*τ*)_mean_ (wt% min^−1^)	△_1/2_ (K)	*D* (wt%^2^ min^−2^ K^−3^)
30	4.49 × 10^−2^	1.57	108	260	1.53 × 10^−1^	3.93 × 10^−2^	218	9.82 × 10^−10^
40	6.41 × 10^−2^	2.24	92	281	2.06 × 10^−1^	7.47 × 10^−2^	384	1.55 × 10^−9^

As shown in table 2, the volatile release parameter of foundry sand increased as the heating rate increased, again indicating that an elevated heating rate benefits the thermal decomposition of foundry sand in high-temperature environments. The volatile release parameter of foundry sand was about one in 1000 of rice straw [23], one in 10 of corncob [25], one in 10 000 of wastewater solids and one in 10 of lignite [30]. The foundry sand is difficult to evaporate at high temperatures compared with other materials, mainly because its volatile content is only about 2.68 wt%. SiO_2_, which is the main component of foundry sand, has high-temperature stability. The analysis results of volatile release parameter were consistent with the proximate and TG/DTG curves analyses.

### 3.4. Thermal decomposition kinetics analysis of foundry sand

**Figure 4.**
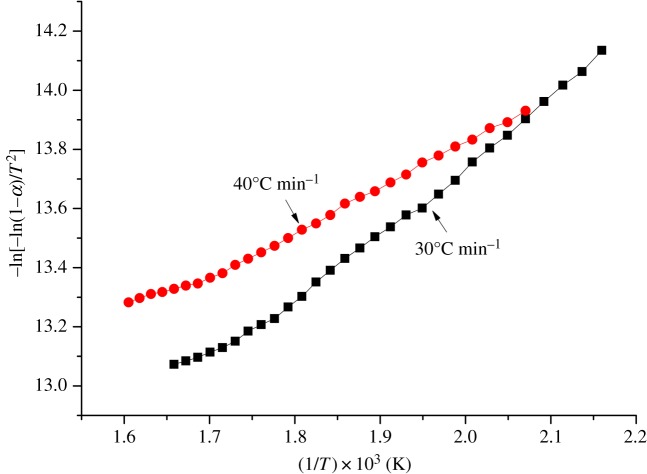
Relationship between −ln[−ln(1 − α)/*T*^2^] and (1/*T*) × 10^3^.

The variations between −ln[−ln(1 − α)/T^2^] and (1/T) × 10^3^ at heating rates of 30 and 40 K min^−1^ in nitrogen are shown in figure 4.

The activation energy *E* and pre-exponential factor *A* were calculated by equation (2.4) as shown in table 3.

**Table 3.** Thermal decomposition kinetics parameters of foundry sand.

*β* (K min^−1^)	temperature range (K)	fitting equation	*E* (KJ mol^−1^)	*A* (min^−1^)	correlation coefficient
30	463–603	*y* = 2.162*x* + 9.4194	17.97	2.61 × 10^−3^	0.9939
40	483–623	*y* = 1.4484*x* + 10.92	12.04	1.05 × 10^−3^	0.9937

In equation (2.4), the temperature range for calculating activation energy and pre-exponential factor should be located in the major mass-loss phase. As shown in figure 2, the temperature ranges from 463 to 603 and 483 to 623 K were the main thermal decomposition phases of the volatiles in the foundry sand at heating rates of 30 and 40 K min^−1^. Therefore, the temperature ranges for the thermal decomposition process were defined. After obtaining the fitting equation of the independent and dependent variables, the fitting effect can be determined by the correlation coefficient. Both correlation coefficients were above 0.99, indicating that the selected temperature range and fitting equation were appropriate.

At a heating rate of 30 K min^−1^, the activation energy of foundry sand for cast iron was about one in four of peanut shell [24], one in four of corncob [25], one in 19 of coal and one in 11 of wastewater solid [30]. Foundry sand has high-temperature stability, as confirmed by the volatile release parameter: a large amount of activation energy is needed to convert volatiles into an active state. The activation energy of foundry sand appeared to be relatively small, which seemingly contradicts the volatile release parameter. This is mainly due to the wide temperature range of thermal decomposition in the foundry sand sample. Most mass loss occurred between 463 and 603 K in this study, which makes a contribution to the total mass loss of about 39 wt%. While the mass loss of corncob in the thermal decomposition zone contributes about 80–90 wt% to its total mass loss [25].

As shown in table 3, the activation energy gradually decreased as the heating rate increased, indicating that the foundry sand easily decomposed, which was consistent with the volatile release parameter analysis results.

## 4. Conclusion

The thermal decomposition characteristics of foundry sand for cast iron were investigated for the first time in this study. A novel method was established to calculate the starting temperature of volatile emission in determining the volatile release parameter of foundry sand and validated by a case study. The conclusions can be summarized as follows.

The thermal decomposition process of foundry sand for cast iron can be divided into three phases. There is no obvious oxidation or combustion reaction of foundry sand in a nitrogen atmosphere, but there is an obvious endothermic peak at about 846 K due to the transition of quartz from *β* to *α* phase.

The thermal decomposition degree of foundry sand increases as the heating rate increases. The peak temperature of the DTG curve also increases with the heating rate due to the temperature gradient between the landmark and the sample, as well as the inside and outside of the sample.

A novel technique to calculate the starting temperature of volatile emission in determining the volatile release parameter of foundry sand was established. Foundry sand does not easily evaporate in high-temperature environments compared to other materials, mainly because its volatile content is only about 2.68 wt%.

The thermal decomposition kinetics parameters of foundry sand, namely activation energy and pre-exponential factor, can be calculated under kinetics theory. The activation energy of foundry sand for cast iron is small compared to other materials, mainly due to the wide temperature range of thermal decomposition in the sand.

## Supplementary Material

Thermal decomposition data

## Data accessibility

The thermal decomposition data that supporting this paper have been uploaded as electronic supplementary material.
